# Arrhythmias Following Patent Foramen Ovale Closure: An Unsolved Enigma

**DOI:** 10.3390/life14121590

**Published:** 2024-12-02

**Authors:** Aikaterini-Eleftheria Karanikola, Stergios Soulaidopoulos, Ioannis Leontsinis, Eirini Dri, Marios Sagris, Athanasios Kordalis, Konstantinos Aznaouridis, Dimitrios Tsiachris, Konstantinos Tsioufis

**Affiliations:** First Department of Cardiology, Hippokration Hospital, Athens Medical School, National and Kapodistrian University of Athens, Vas. Sofias 114, 11527 Athens, Greecedtsiachris@yahoo.com (D.T.);

**Keywords:** patent foramen ovale, occlusion, supraventricular arrhythmia, atrial fibrillation, inflammation

## Abstract

Patent foramen ovale (PFO) closure has proven to be an effective method of reducing the risk of recurrent stroke in patients with embolic stroke of unknown origin (ESUS). One of the most recognized post-procedural complications is the de novo occurrence of supraventricular arrhythmias, mainly atrial fibrillation, in the first three months following PFO closure. Earlier studies reported the incidence to be around 3.4–7%; however, this percentage has risen in recent studies up to 21%. The pathogenesis behind this type of arrhythmia is complex and not clearly understood, although it seems that direct effects of the device on the atria, as well as an inflammatory response, are the two most prevalent mechanisms. Management of this complication might be challenging given the heterogenicity of patient characteristics, so an individualized approach is most wisely followed. This review aims to present the current data on the incidence, pathogenesis and therapeutic strategies behind this rather common concern in an era of increasing transcatheter interventions for PFO.

## 1. Introduction

Supraventricular arrhythmias, particularly atrial fibrillation, represent a rather frequent complication following transcatheter patent foramen ovale (PFO) closure. Considering that PFO closure aims to minimize the risk of recurrent stroke in individuals with cryptogenic stroke, the occurrence of supraventricular arrhythmias, more commonly in the early post-implantation phase, can perpetuate concerns about the hazard of experiencing a new embolic event and raise uncertainties about the overall benefits of the intervention. Furthermore, AF presentation after PFO closure can be rather disturbing, especially in cases when older age and comorbidities challenge the index stroke causality [1]. Anticoagulation need and its duration can arbitrarily rely on existent stroke risk scores [2]. On the other hand, the precise risk for developing AF in the long-term after PFO closure remains for the moment unknown [3]. In this regard, the clinical significance and the necessity to treat or even prevent these procedure-related arrhythmias remain to be conclusively established.

The mechanisms contributing to arrhythmogenesis are still not fully understood. Among others, a possible explanation is that mechanical forces exerted on the myocardial tissue, related to the implanted device, promote a localized inflammatory reaction at the interface between the device and the atrial septum. This inflammatory response is likely to play a key role in the substrate formation of atrial arrhythmias [4]. To that end, the peri-procedural use of anti-inflammatory or antiarrhythmic agents could theoretically constitute a promising approach; however, evidence in this direction is scarce. Consequently, there is a lack of effective measures to prevent the onset of atrial arrhythmias after transcatheter PFO closure, which is a bit of a paradox considering the widespread application of the technique over the last decades.

Within this context, the aim of this article is to summarize the emerging evidence on the incidence and pathogenesis of supraventricular arrhythmogenesis after PFO closure and to explore potential therapeutic strategies.

## 2. Incidence of Arrhythmias

The incidence rates of postprocedural AF vary among different trials. More specifically, in the six major trials on PFO closure conducted between 2000 and 2017 (Closure, PC Trial, Respect, Close, Reduce, Defense-PFO), the cumulative incidence was 3.2%, with more than three-quarters of the episodes being detected in the first 45 days after the procedure [5]. Most investigators agree that the choice of percutaneous PFO closure compared to medical therapy, such as antiplatelets and oral anticoagulants, is accompanied by a five-fold increase in the risk of new-onset AF [6,7]. Surgical PFO closure also seems to increase the risk of postprocedural AF by 4.9 times [8]. The incidence of atrial arrhythmias may be even higher in this group of patients, as data from studies using extensive rhythm monitoring with external or implantable loop recorders suggest, with AF detection rates being somewhere between 14% and 21% [9,10,11].

Different devices have been linked to variable rates of supraventricular arrhythmias. There are five occluder types currently approved for PFO closure. The only ones carrying both an FDA approval and a CE mark are the Amplatzer PFO Occluder (Abbott Laboratories, Abbott Park, IL, USA) and the Gore Cardioform Septal Occluder (W.L. Gore and Associates, Newark, DE, USA). Both have double-disk design; however, several meta-analyses comparing them agree that the rate of postprocedural AF is lower for the Amplatzer device [12,13]. This is in concordance with the results from the Respect and Reduce randomized clinical trials that reported rates of AF around 1.2% for the Amplatzer and 6.6% for the Gore Occluder, respectively [14,15]. In addition, AF rates were higher with the older, bulkier, double-umbrella-like occluders (CardioSEAL, Starflex, Seoul, Republic of Korea) which are no longer available^6^. Currently, some devices on the market are manufactured with softer materials in order to reduce mechanical stress on the atria and are covered by a wire net in order to minimize nickel release to the bloodstream. The Cocoon PFO Occluder (Vascular Innovations Co., Ltd., Khlong Khoi, Thailand, CE mark)—a softer, platinum-nanocoated device—reported a 1.6% rate of postprocedural atrial fibrillation/flutter and 2.6% for minor atrial arrhythmias in a 3.5-year follow-up, which is one of the lowest reported rates [16,17,18]. Similar devices, such as the Ceraflex PFO Occluder (Lifetech Scientific, Shenzhen, China) and the Figulla Occlutech system (Occlutech GmbH, Jena, Germany) with titanium nitride and titanium oxide coating, respectively, observed a yearly incidence of SVTs or AF of 4–5% [19,20]. Rates of AF/SVT occurrence across different devices for PFO closure are shown in Table 1. 

## 3. Predictors of Arrhythmias

Patient-related parameters are the first contributing factors to be considered in the development of postprocedural supraventricular arrhythmias. Older age, particularly > 60 years, has been identified as one such predictor, also reflecting the increased risk of AF with advancing age in the general population [4,21]. Diabetes has also been linked with increased AF risk in this group of patients [11,21]. In a subgroup analysis of the Reduce trial, male sex appeared to be a statistically significant factor for postprocedural AF development, with device size and age showing a weaker link [22].

In addition, pre-closure echocardiographic features have been recognized as important indicators of patients at risk for postprocedural AF. Increased left atrial size in PFO patients who have suffered a cerebrovascular event is correlated with higher rates of arrhythmias after closure [23]. Bonvini et al. highlighted that high-risk PFO anatomy—such as a large PFO and the presence of an atrial septal aneurysm (ASA), as detected with echocardiography—also predisposes towards AF occurrence [24]. Residual shunt post-closure increased the frequency of AF development in one report; however, these data require validation with larger series [25].

It is not only the design features but also the size of different devices that has been mentioned as a predictor of atrial tachyarrhythmias. Alaeddini et al. correlated the incidence of atrial tachyarrhythmias to a device size of 33 mm, while novel reports suggest that a left disk diameter of more than 29 mm is an important arrhythmogenic factor to consider [9,25,26].

Finally, the hypothesis of atrial vulnerability—as illustrated by atrial refractory period measured during an electrophysiologic study and inducibility of AF—in patients with atrial septal abnormalities, such as PFO, ASD and ASA, has been proposed as a potential risk factor for AF development in up to 50% of cases [27,28]. It seems that pre-closure atrial remodeling secondary to anomalous atrial anatomy promotes AF development post-closure, and this effect is further exacerbated the longer the septal defect remains unrepaired [29].

## 4. Mechanisms of Atrial Arrhythmias Following Percutaneous PFO Closure

Regardless of the aforementioned patient- and device-related predictors, it is well established that the risk of arrhythmias is higher in the immediate period after PFO closure. Most studies used as cut-off points for the follow-up the first 45 days, 3 months or 6 months after the procedure. A Danish cohort reported that AF/atrial flutter rates beyond the first 90 days after PFO closure are no greater that those occurring in medically-managed PFO [3]. This fact further supports the hypothesis that mechanical irritation of the atria, as well as inflammation, may contribute to increased arrhythmogenicity post-occluder device implantation [30].

The mechanical implications of an interatrial septum device and the subsequent left atrial remodeling are represented in most studies by speckle tracking echocardiographic (STE) LA strain measurements during the three atrial phases (contractile, reservoir, conduit). It has been shown that in the immediate 3-month follow-up period, contractile LA strain of the anterior wall is reduced, which—combined with an increase in LA volume— may contribute to higher incidence of PAF. In one study, these alterations were balanced by an increase in the strain of the lateral LA wall, and returned to baseline in the 6-month follow-up period [31]. Similar results are provided by Qiu et al., with LA reservoir and conduit strain recovering within 3-6 months of the procedure, while contractile strain may remain decreased for up to a year [32].

Vitarelli et al. studied patients pre- and 24 h post-PFO closure, measuring both 2D-STE deformation and 3D-STE volumetric parameters represented by time-to-peak strain (TPS) and expansion index (EI), respectively. A decline in peak left atrial strain after device implantation in all LA segments, particularly the middle septal ones—coinciding with the area of the device—was observed. Furthermore, both 2D-STE LA-TPS and 3D-STE EI were independent predictors of paroxysmal atrial fibrillation in a 6-month follow-up period [33].

Elevation in the LV filling pressures, as represented by a decrease in LA reservoir GLS and an increase in LA volume index and E/e’ ratio, has also been observed in the first 24 h following PFO closure [34]. These observations have not yet been adequately understood. One explanatory mechanism could be related to the shunt closure. In fact, the opposite principle is being tested as a treatment method in heart failure with preserved ejection fraction (HFpEF), where an intra-atrial shunt is created to relieve the abnormally elevated left atrial pressure towards the right circulation. In the case of PFO closure, the deletion of a pre-existing trivial left to right shunt could contribute to higher left atrial pressures, especially in circumstances of lower atrioventricular compliance [35]. On the other hand, the abovementioned mechanical atrial wall compression may give rise to oedema and local inflammation contributing to atrial arrhythmias.

Apart from the mechanistic effects of the device, systemic and local inflammation has been a long-identified causative factor in the pathogenesis of atrial fibrillation, as it leads both to electrical and structural remodeling of the atria [36]. After implantation of an intracardiac device, a foreign-body reaction is observed. This includes the pro-inflammatory phase, which is driven by necrotic myocardial cells and results in the formation of the inflammasome; the cell proliferation phase, which includes further activation of inflammatory cytokines and growth factors; and finally, the endothelization or resolution phase [37]. Many of these inflammatory molecules—with TNF-a and IL-6 being the most frequently studied ones—have posed a strong link with de novo atrial fibrillation, particularly after procedures such as cardiac surgery or catheter ablation [38]. Their arrhythmogenic effects include promotion of cardiac fibrosis, gap junction impairment, alterations in calcium handling and sympathetic nervous system overactivation, all of which may induce cardiac arrhythmias via the three most established mechanisms of reentry, triggered activity and automaticity [39]. Johnson et al. have also suggested that inflammation and the fibrous tissue surrounding the device may alter interatrial conduction and promote remodeling and arrhythmias, as depicted by electrocardiographic changes in P-wave duration [40].

Apart from the reaction to tissue injury, allergic reactions to materials commonly used in the coating of intracardiac devices have been investigated as potential allergens, thus triggering the inflammatory cascade and potentially increasing the arrhythmic burden. Nitinol, a mixture of nickel and titanium, constitutes one of the most commonly used coating materials of PFO occluders, as well as being a known allergen in up to 19% of adult population [41,42,43]. After nickel contact, a type IV hypersensitivity reaction may occur. This is a T-cell-mediated, delayed inflammatory reaction with activation of dendritic cells, effector T-cells and cytokines, such as TNF-a and ILs, and should be taken into consideration when assessing patients with postprocedural arrhythmias [44]. Finally, although there have been few mentions of anaphylaxis-induced atrial fibrillation, owing to the effects of histamine on myocardial cells, the risk of type I reactions to metal implants remains extremely low [45,46].

Finally, Rigatelli et al. propose two more pathogenetic mechanisms of postprocedural atrial arrhythmias. The first one suggests that the device may act as an electrical obstruction, thus giving rise to macro-reentrant circuits, and the second one investigates the possibility of antiplatelet-related gastric injury in increasing extrasystolic beats [47].

## 5. Therapeutic Considerations

As far as the management of arrythmias occurring post-PFO closure, an individualized approach should be followed, taking into consideration the timing of presentation, the disease burden and, of course, patient-related characteristics and risk factors for adverse effects and, most importantly, systemic thromboembolism.

A large meta-analysis of 8 controlled trials and 16 cohort studies concluded that about 25% of new-onset AF converted spontaneously to sinus rhythm, 65% was referred for rhythm control via electrical or pharmacological cardioversion, 2% required catheter or surgical ablation and, for the remaining percentage, a rate-control strategy was decided. Of those patients, 41% were placed under systemic anticoagulation, with nearly three-quarters requiring long-term antithrombotic therapy beyond 6 months [6]. A therapeutic algorithm for AF related to PFO closure is proposed by Elgendy et al. The authors distinguish AF as primary or secondary based on late—that is, occurring >45 days post-procedure—and early occurrence, respectively. In the second case, especially if cardioversion to sinus rhythm is achieved in less than 48 h, they are more latent in administering long-term anticoagulation therapy [48]. However, a gap of evidence from current AF guidelines as to the management of secondary AF still remains [49,50].

Given the timing of most arrhythmias in the acute phase and the small burden of the disease, few patients are likely to be candidates for rhythm control through interventional ablation techniques. Nevertheless, when required, special maneuvers for accessing the left atrium through the native septum or through the occlusion device have been described [29,51,52]. Transesophageal or intracardiac echocardiography guidance during the procedure may also assist in locating the optimal transeptal puncture site, although use of pre-procedural CT has also proven extremely helpful in imaging the exact anatomical structures and in planning all technical aspects [53].

Apart from treating arrhythmias when they occur, a different approach of targeting their pathogenetic background, and thus preventing their development, may be followed. In this context, a potential pharmacological goal could be reducing the postprocedural inflammation related to AF. Various researchers now focus on investigating the effect of leucocyte-secreted cytokines on cardiomyocytes and their conduction properties, shedding light onto potential immunologic targets for atrial fibrillation management [54,55]. Current anti-inflammatory agents, such as statins, corticosteroids and colchicine, have been utilized in the prevention of postoperative and post-pulmonary vein isolation atrial fibrillation. Among those, colchicine has shown the most promising results [56,57,58,59]. So far, none of these agents have been investigated in the setting of transcatheter procedures.

Finally, the administration of antiarrhythmic agents for the prevention of new-onset AF in patients at increased risk has been studied with beta-blockers and Amiodarone in cardiac surgery patients and is even guideline-recommended [50]. Currently, the role of Flecainide in reducing the development of atrial arrhythmias in patients undergoing PFO closure is being studied [60].

## 6. General Considerations

The diagnosis of AF detection of post-PFO closure is expected to rise in the era of implantable loop recorders and smart devices. These events are asymptomatic in almost half the patients. As previously mentioned, the few studies to date to monitor patients post-closure with insertable cardiac monitors (ICMs) report a rate of supraventricular arrhythmias as high as 20.9% in the first month after the procedure [9]. Extensive rhythm monitoring may also be useful pre-PFO closure. Various studies have confirmed that supraventricular arrhythmias are higher among this population, owing to increased atrial vulnerability and morphological changes in the atrial anatomy due to presence of a shunt. This pathological atrial activity may play a role in the embolic source of the stroke in patients with atrial septal abnormalities [28]. Considering that most patients referred for PFO closure have suffered from embolic events, postprocedural atrial fibrillation may question the true etiology of the index stroke. In cases where initial testing post-stroke with a 12-lead ECG and 24 h Holter monitoring is not diagnostic for AF, current guidelines recommend monitoring with an ICM for up to 6 months, especially in patients with concomitant AF risk factors, before characterizing the cerebrovascular event as PFO-related [12]. According to the results of the Crystal AF trial, the detection of AF in embolic stroke of unknown source (ESUS) patients was 6 times higher in the ICM group vs. the control group, highlighting the potential role of extensive rhythm monitoring in such patients [61].

Another point to consider is choosing the right occlusion device, not only based on the anatomical characteristics of the PFO but also the patient’s arrhythmic risk profile. As previously mentioned, older, male patients that are already at increased danger of new-onset AF post-closure may be more suitable candidates for novel, softer devices that cause less mechanical stress on the atria. However, this point remains yet to be validated by larger trials.

Rates of recurrent cerebrovascular events post-PFO closure remain low—approximately 1% to 4% during long-term follow-up; however, the exact percentage related to new-onset AF has not been yet confirmed by large trials [62,63,64]. A large cohort studied 515 patients for a follow-up of 11 years post-PFO closure and reported a rate of 7.8% for recurrent cerebrovascular events, with more than half of them being related to atherosclerotic disease. The rate of de-novo AF was 5%; however, cardioembolic events occurred in only 8.8% of the stroke group [65]. Another retrospective cohort with a mean follow-up of 7 years reported similar rates of recurrent all-cause stroke, with 70% being non-cardioembolic and only 15% being AF-related. Interestingly, those with a new diagnosis of AF were older at the time of PFO closure and had at least one of the classic predisposing AF factors, such as hypertension [66].

What remains yet to be addressed by large trials is the residual stroke risk related to AF in the immediate phase after the procedure, in order to further clarify the clinical relevance of these supraventricular arrhythmias. Until robust data on these issues are presented, the appearance of this rather frequent postprocedural complication should in no way act as a hindrance in opting for PFO closure, given the established clinical benefit of this transcatheter intervention.

## 7. Conclusions

In the era of transcatheter interventions, PFO closure for secondary prevention of embolic stroke is gaining ground against medical therapy, owing to its superior results. Supraventricular arrhythmias in the immediate postprocedural period have been recognized as one of the major complications of the procedure, although their true incidence remains a subject of discussion and highly depends on the timing and method of screening. Future research should focus on clarifying the potential pathogenesis and risk factors behind these arrhythmias, as well as their clinical significance and optimal management.

## Figures and Tables

**Table 1:** Rates of AF/SVTs across currently available PFO occluders.

Device	Design	AF/SVT Incidence
Amplatzer PFO Occluder [14]	Double-disk/nitinol wire	1.2%
Gore Cardioform Septal Occluder [15]	Double-disk/nitinol wire	6.6%
Cocoon PFO Occluder [16,17,18]	Double-disk/platinum-nanocoated nitinol wire	1.6%
Ceraflex PFO Occluder [19]	Double-disk/nitinol wire with titanium nitride	4–5%
Figulla Occlutech system [20]	Double-disk/nitinol braiding with titanium oxide	4–5%
